# Sarcorucinine-D Inhibits Cholinesterases and Calcium Channels: Molecular Dynamics Simulation and In Vitro Mechanistic Investigations

**DOI:** 10.3390/molecules27113361

**Published:** 2022-05-24

**Authors:** Asaad Khalid, Mohnad Abdalla, Maria Saeed, Muhammad Nabeel Ghayur, Surya Kant Kalauni, Mohammed Albratty, Hassan A. Alhazmi, Mohammed Ahmed Mesaik, Anwarul Hassan Gilani, Zaheer Ul-Haq

**Affiliations:** 1 Substance Abuse and Toxicology Research Center, Jazan University, P.O. Box 114, Jazan 45142, Saudi Arabia; haalhazmi@jazanu.edu.sa; 2 Medicinal and Aromatic Plants and Traditional Medicine Research Institute, National Center for Research, P.O. Box 2424, Khartoum 11111, Sudan; 3 Key Laboratory of Chemical Biology (Ministry of Education), Department of Pharmaceutics, School of Pharmaceutical Sciences, Cheeloo College of Medicine, Shandong University, 44 Cultural West Road, Jinan 250012, China; mohnadabdalla200@gmail.com; 4 Dr. Panjwani Center for Molecular Medicine & Drug Research, University of Karachi, Karachi 75530, Pakistan; mariyasaeed@yahoo.com (M.S.); zaheer_qasmi@hotmail.com (Z.U.-H.); 5 Department of Biomedical Sciences, University of Pikeville, Pikeville, KY 41501, USA; nabeelghayur@yahoo.com; 6 Department of Biological and Biomedical Sciences, Aga Khan University, Karachi 74800, Pakistan; vc@uoh.edu.pk; 7 Central Department of Chemistry, Tribhuvan University, Kirtipur 44618, Nepal; skkalauni@gmail.com; 8 Department of Pharmaceutical Chemistry, College of Pharmacy, Jazan University, P.O. Box 114, Jazan 45142, Saudi Arabia; malbratty@jazanu.edu.sa; 9 Department of Medical Microbiology, Faculty of Medicine, University of Tabuk, Tabuk 71491, Saudi Arabia; mmesaik@ut.edu.sa; 10 Department of Public Health and Nutrition, University of Haripur, Haripur 22620, Pakistan

**Keywords:** acetylcholinesterase, butyrylcholinesterase, steroidal alkaloids, inhibition kinetics, molecular dynamics simulation, ligand-protein docking, calcium channel blocker

## Abstract

Acetylcholinesterase (AChE) inhibitors and calcium channel blockers are considered effective therapies for Alzheimer’s disease. AChE plays an essential role in the nervous system by catalyzing the hydrolysis of the neurotransmitter acetylcholine. In this study, the inhibition of the enzyme AChE by Sarcorucinine-D, a pregnane type steroidal alkaloid, was investigated with experimental enzyme kinetics and molecular dynamics (MD) simulation techniques. Kinetics studies showed that Sarcorucinine-D inhibits two cholinesterases—AChE and butyrylcholinesterase (BChE)—noncompetitively, with K_i_ values of 103.3 and 4.66 µM, respectively. In silico ligand-protein docking and MD simulation studies conducted on AChE predicted that Sarcorucinine-D interacted via hydrophobic interactions and hydrogen bonds with the residues of the active-site gorge of AChE. Sarcorucinine-D was able to relax contractility concentration-dependently in the intestinal smooth muscles of jejunum obtained from rabbits. Not only was the spontaneous spasmogenicity inhibited, but it also suppressed K^+^-mediated spasmogenicity, indicating an effect via the inhibition of voltage-dependent Ca^2+^ channels. Sarcorucinine-D could be considered a potential lead molecule based on its properties as a noncompetitive AChE inhibitor and a Ca^2+^ channel blocker.

## 1. Introduction

Alzheimer’s disease (AD) is a chronic and progressive neurodegenerative condition that causes the decline of intellectual functioning in various areas of the brain in aged people [1]. This disease, which affects more than 18 million people worldwide, is histopathologically characterized by the development of neurofibrillary tangles and amyloid plaques [2]. The tangles are generated from microtubular hyperphosphorylated tau protein accumulation, whereas the senile plaques are complex extracellular lesions mainly composed of β-amyloid (βA) peptide aggregates [2]. The current leading therapy for AD is the inhibition of the acetylcholinesterase (AChE) enzyme, with mainly cognitive symptomatic effects. AChE inhibitors are prescribed to patients in the mild and moderate stages of AD and effectively manage neuropsychiatric symptoms [3,4,5]. However, substantial dose-dependent adverse effects make it difficult to employ these currently available cholinesterase inhibitors (ChEIs). The short half-life of several ChEIs, such as rivastigmine and physostigmine, jeopardizes their long-term therapeutic usage, in addition to their respective adverse effects. Synthetic analogs of classical ChEIs, such as ladostigil, tacrine, and indenyl derivatives, have fewer adverse effects, but their effectiveness is restricted by their inability to pass the blood-brain barrier. To overcome the limitations of traditional ChEIs and contribute to the enhancement of existing AD therapies, the development of innovative synthetic ChEIs with enhanced pharmacokinetic characteristics and potency remains a top objective. 

The cholinesterases (ChEs) are a family of two related enzymes, as follows: AChE (EC 3.1.1.7) and butyrylcholinesterase (BChE, EC 3.1.1.8) [6]. The active site of ChEs is buried slightly above the bottom of a deep gorge that penetrates midway into these enzymes. Fourteen highly conserved aromatic amino acids line approximately 40% of the active-site gorge surface. The active-site gorge of human and *Torpedo californica* AChE consists of four subsites that are involved in molecular recognition and catalysis, as follows: (1) an acylation site near the bottom of the gorge; (2) a peripheral anionic site (PAS) at the top of the gorge; (3) the esteratic locus, which consists of two domains—the active site catalytic triad and the so-called oxyanion hole; and (4) the quaternary ammonium-binding locus [7]. The overall structure of BChE closely resembles that of humans and *Torpedo* AChE [8]. However, the significant differences between the two enzymes are as follows: (1) Several aromatic residues lining the gorge of the AChE are replaced with hydrophobic analogs in the gorge of BChE; (2) the acyl-binding pocket of the BChE gorge contains Leu286 and Val288, instead of the Phe288 and Phe290 in AChE, enabling BChE to accommodate bulky ligands in its relatively large gorge; (3) the conformation of the acyl pocket is different in the gorges of both enzymes. Moreover, BChE and chicken AChE lack Trp279 necessary for binding bis-quaternary ligands at the PAS [9].

Pregnane-type steroidal alkaloids have many pharmacological properties, including antitumor, ganglion-blocking, and blood pressure-lowering effects [10]. We have previously reported the ChE inhibitory properties of many pregnane-type steroidal alkaloids isolated from various medicinal plants [11,12,13,14,15,16,17,18].

In recent years, the use of molecular dynamics (MD) simulations in molecular biology and drug discovery has skyrocketed. MD simulations have aided in the drug discovery process i.e., conformational analysis of protein–ligand complexes and cryptic or allosteric protein binding sites, the improvement of classic virtual screening approaches, and the prediction of ligand binding energies directly [19,20]. Current experimental approaches make the understanding of the atomistic energetics and mechanics of binding impossible since ligand binding and the essential macromolecular movements that go along with it are microscopic events that happen in millionths of a second. We believe that combining MD simulations with complementary experimental approaches is a viable tool for studying the characteristics and dynamic behavior of proteins associated with inhibitor and activator complexes, which will be beneficial in neuroscience and beyond. Effectively simulating molecular biology and drug development requires the careful evaluation of both experimental and computational data, necessitating broad expertise and multidisciplinary collaboration [19,20,21].

In the current study, the molecular basis of ChE inhibition by natural Sarcorucinine-D [14,15] was investigated through experimental enzyme inhibition kinetics and computational insights, via molecular docking and molecular dynamics simulation approaches. The current study was conducted on *Torpedo* AChE (which shares more than 70% structural homology with human AChE) and horse serum BChE (which shares approximately 90% of its amino acid sequence with human BChE). In silico docking and MD simulations were conducted on AChE, being the primary drug target for anti-Alzheimer’s drugs. AChE inhibitors are known to boost cholinergic neurotransmission; hence, the action of Sarcorucinine-D on isolated rabbit jejunum tissues and Ca^2+^ channels was also investigated. The cytotoxic effects of Sarcorucinine-D were assessed on Madin–Darby bovine kidney (MDBK) cell proliferation.

## 2. Results and Discussion

### 2.1. Inhibition Kinetics of AChE and BChE

The pregnane-type steroidal alkaloid Sarcorucinine-D has a basic steroidal skeleton with a dimethylamino substituent at C-20 and a hydroxyl moiety at the C-3 position (the structure of Sarcorucinine-D is shown in Table 1).

**Figure 1:**
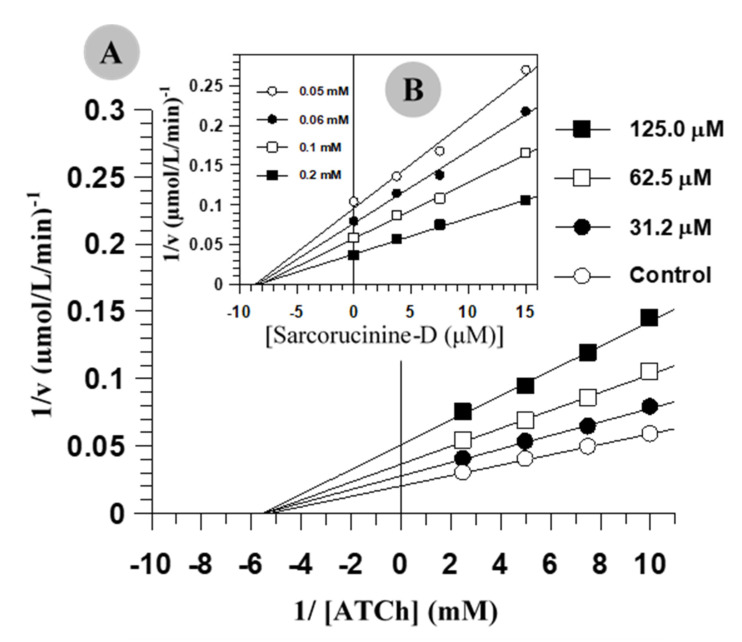
Steady-state inhibition of ChEs by sarcorucinine-D. (**A**) *Tc*AChE inhibition: Lineweaver–Burk plot of reciprocal of the initial velocities versus reciprocal of ATCh in the presence of various concentrations of inhibitor. (**B**) BChE inhibition: Dixon plot of reciprocal of the initial velocities versus various concentrations of sarcorucinine-D.

The kinetic parameters of AChE and BChE inhibition by Sarcorucinine-D are summarized in Table 1. Sarcorucinine-D exhibited a noncompetitive type of inhibition against AChE and BChE, as it decreased the maximum velocity (V_max_) without affecting the enzyme’s affinity for the substrate (K_m_). The purity of the noncompetitive inhibition was predicted from the secondary replots of Lineweaver–Burk plots, which showed linear lines rather than hyperbolic lines, suggesting partial noncompetitive inhibition. Figure 1 shows the graphical analysis of the steady-state inhibition of AChE and BChE by sarcorucinine-D.

The noncompetitive mechanism of inhibition exhibited by Sarcorucinine-D implies that this compound does not interact with the catalytic triad of the active site of any ChE. However, the compound may bind at any other subsite of the active-site gorge of the enzymes. The alternative possibility of off-gorge binding, which may require massive conformational changes in the structure of the enzymes, was unlikely—as inferred from the linear progress of the enzyme-catalyzed reactions in the presence of various concentrations of this compound (data not shown). Molecular dynamics simulations show that the interactions of similar steroidal alkaloids with AChE do not change the structure of the gorge of the enzyme, but do have a significant effect on the dynamics of the gorge width [22].

The data presented in Table 1 show that Sarcorucinine-D is approximately 22 times more selective toward the BChE. This selectivity could be caused by the sizeable active-site gorge of BChE, enabling the compound to diffuse easily and efficiently rotate inside the gorge for a good fit. 

Sarcorucinine-D was a selective ChE inhibitor when screened for the inhibition of phosphodiesterase, urease, β-glucuronidase, and α-glucosidase enzymes.

The results of this study and our previous inhibition kinetic, docking [22,23], and CoMFA [12] studies conducted on structurally similar compounds suggest that the competitive inhibition of Sarcorucinine-D with AChE or BChE is highly unlikely. This finding could be partly due to the bulky hydrophobic structure of sarcorucinine-D, which is substantially different from the cationic aliphatic substrate acetylcholine and butyrylcholine.

### 2.2. Molecular Docking Investigations against AChE

**Figure 2:**
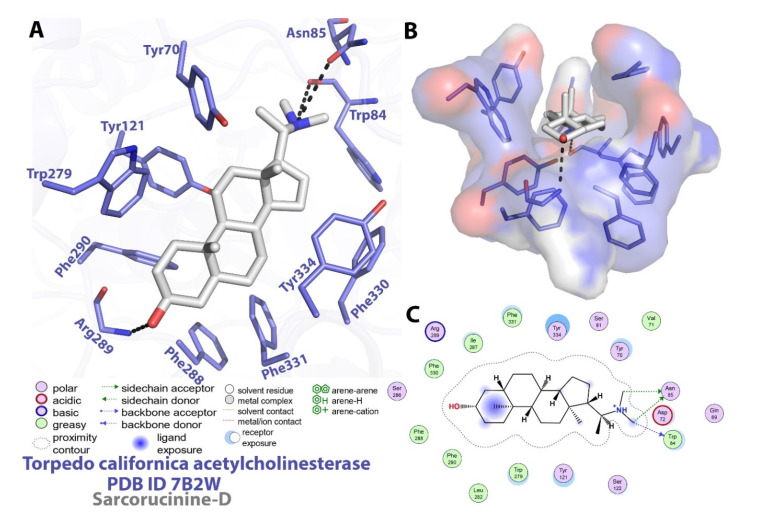
Post-docking analysis of *Torpedo californica* acetylcholinesterase with sarcorucinine-D. (**A**) Stereoview of the interactions of amino acid residues with sarcorucinine-D (**B**) Top surface view showing the orientation of sarcorucinine-D inside the active-site gorge of AChE (**C**) Detailed interactions of sarcorucinine-D and amino acid residues.

Due to its clinical importance as a drug target against Alzheimer’s disease, a molecular inspection of the executed docking output of Sarcorucinine-D against AChE was utilized to interpret the binding mode analysis. The Auto-Dock topmost-ranked output posited a −12.73 kcal/mol binding energy score, which was further utilized for molecular interaction profiling. The interactions of Sarcorucinine-D with AChE are listed in detail in Table 2 and are shown graphically in Figure 2. 

Our previous studies predicted that the six-membered ring A of a similar type of steroidal alkaloids first entered the active-site gorge and placed the C-3 functional groups at the bottom of the gorge [11,22,23]. Sarcorucinine-D also penetrated the gorge with its six-membered ring (ring A), placing the C-3 hydroxyl group at the bottom of the AChE gorge (Figure 2).

In addition to the critical hydrogen bonding observed between Sarcorucinine-D and Trp84 and Asn85 (depicted in Figure 2), hydrophobic interactions are apparently the most prominent stabilizing forces in the AChE–Sarcorucinine-D complex. Table 2 lists the non-polar (hydrophobic) contacts between Sarcorucinine-D and various residues of the active-site gorge of AChE. The docked pose demonstrated that the giant hydrocarbon skeleton of Sarcorucinine-D established hydrophobic contacts with the aromatic amino acid residues of AChE of the peripheral anionic site (Tyr70, Tyr121, Trp279, Tyr334), an anionic subsite (Trp84, Phe330), an anionic catalytic site (Asn85, Phe331), and acyl pocket residues (Phe288, Arg289, Phe290). These interactions are shown in Figure 2.

Modeling experiments showed that the critical residues at the catalytic triad of AChE, such as Ser200 and His440, did not significantly interact with sarcorucinine-D. Conversely, they had some destabilizing (hydrophobic–hydrophilic) contacts with the inhibitor (Table 2). The prediction of the LPC software [25] supported the inhibition kinetic data that showed Sarcorucinine-D as a noncompetitive inhibitor of AChE.

### 2.3. MD Analysis of Sarcorucinine-D in the Vicinity of AChE

The Molecular Dynamic Simulation interpretation parameters included elemental quantitative analysis to compute Sarcorucinine-D complex constancy in terms of stability, alterations, deviations, and fluctuation of the protein and ligand, in order to check conformational differences during 100 ns of a production run.

### 2.4. AChE Deviation and Fluctuation in the Context of Sarcorucinine-D

The widely used molecular dynamics simulation parameter root mean square deviation (RMSD) was used to examine the displaced atoms of the Sarcorucinine-D–AChE complex during an MD production run time of 100 ns. The deviations were recorded against the C-α backbone atoms of the AChE and the heavy atoms of the Sarcorucinine-D utilized in the simulation.

**Figure 3:**
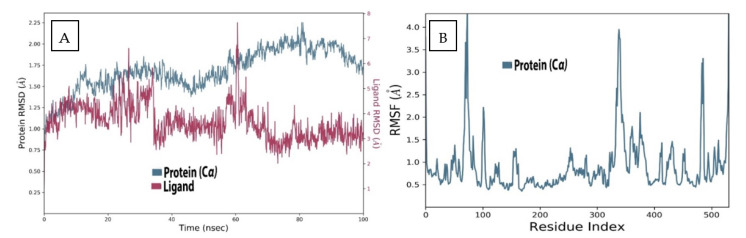
Deviation and fluctuation analysis in the AChE–Sarcorucinine-D complex. (**A**) RMSD of the backbone carbon-alpha atoms of the protein and ligand heavy atoms (**B**) RMSF plot of the protein–ligand complex. Blue color indicates the alpha-carbon backbone of the protein while the maroon color indicates the ligand.

The RMSD analysis of the AChE receptor with Sarcorucinine-D complex, as depicted in Figure 3A, illustrated that AChE receptors underwent deviations with an interval difference of 0.9Å to 2.25Å throughout the projected MD run of 100 ns. Extensive deviations were noted between 40 ns to 80 ns, with a deviation of ±1Å from 1.25Å to 2.25Å. Despite this, the ligand-fitted RMSD of Sarcorucinine-D heavy atoms in AChE receptors displayed more exaggeration in the initial 25 to 60 ns of the projected MD production run—within the range of 0.75Å to 7.5Å—while stability was almost acquired after 80 ns to 100 ns, with a minimum deviation of ±3.5Å. Large deviations were noted simultaneously, precisely at 60 ns for both the ligand and protein. The Lig fit Prot plot revealed that Sarcorucinine-D extended away from the binding region. The root mean square fluctuation of the AChE–Sarcorucinine-D complex with 537 amino acid residues was also computed. Fluctuation differences were spotted with the following amino acid residues: Tyr70, Trp84, Tyr121, Trp279, Phe288, Phe331, and Tyr334. A significant fluctuation was noted at the Tyr70 and Phe330–Tyr334 amino acid residues of the PAS, as shown in Figure 3B. Minor fluctuations were also recorded with the other interacting PAS and an anionic subsite region. The lower deviation-in terms of the RMSD and an acceptable fluctuation in RMSF values- suggested that AChE, when complexed with Sarcorucinine-D, showed stable results in the vicinity of the binding groove.

### 2.5. Protein–Ligand Interaction Contact Analyses

**Figure 4:**
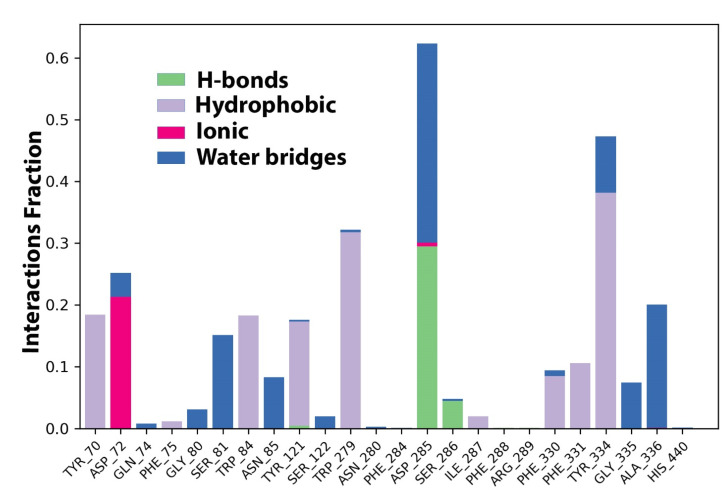
Histogram analysis of the interacted fraction pattern of amino acid residues with the Sarcorucinine-D.

The AChE–Sarcorucinine-D stability was further affirmed by estimating protein–ligand contact histogram bar plots to conceal the overall intermolecular contacts with the active site’s crucial amino acid residues in order to address the interaction fraction patterns. These contact analyses were also estimated on the projected simulation trajectories of a 100 ns MD production run. The intermolecular interaction patterns focused on hydrogen bonding, hydrophobic bonds such as pi–pi, pi-cation interactions, and ionic and water-bridging interactions. Hydrogen bonding interactions have an essential role in assisted computer-aided drug design, and during the analysis, we obtained a hydrogen bonding occupancy of 0.28% at Asp285, 0.6% at Ser286, and <0.1% at Tyr121. Hydrophobic contacts with Tyr70, Trp84, and Tyr121 were at 1.8%, while Trp279 showed 0.35%, Phe330 and Phe331 had a deviation of ±0.1%, and lastly, Tyr334 showed 0.4% contacts, respectively. Phe75 showed a low interaction hydrophobic pattern of <0.1% during the 100 ns MD run. Ionic bonding was also observed at Asp72, with an occupancy of 0.2%. Some water-mediated bridging contacts were also seen with Asp72, Gln74, Gly80, Ser81, Asn85, Ser122, Ser286, Phe330, Tyr334, Gly335, and Ala336. Asp285 showed a maximum 33% H-bond occupancy, which was converted into water bridging-mediated interactions; upon reaching 100 ns simulated time, the interaction had dissolved. A 2D protein–ligand interaction pattern was also checked to provide further information, as depicted in Figure 4.

**Figure 5:**
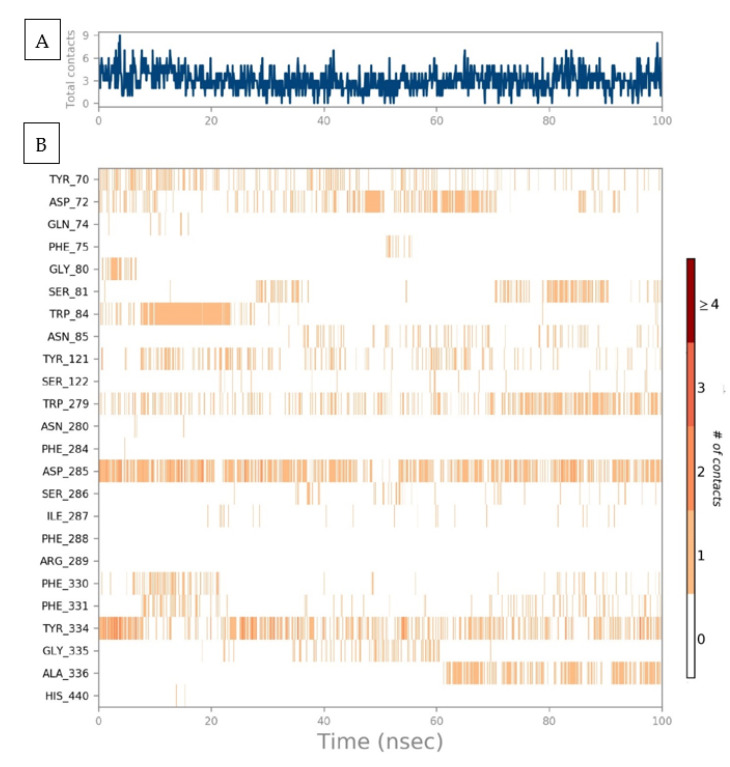
(**A**) A timeline representation of the total no. of molecular interaction contacts in each trajectory frame and (**B**) the no. of interactions with the active site residues in each frame of the 100 simulated trajectory frames.

A timeline representation of the AChE–Sarcorucinine-D histogram was examined to compute the total number of interaction contacts in every frame of the 100 ns projected trajectories. A top panel represented eight to nine molecular interactions at different points in the simulated trajectories depicted in Figure 5A. For a clearer picture, a second timeline representing interacting amino acids that established molecular contacts that persisted during the whole MD run was taken into account, as illustrated in Figure 5B; the amino acids that showed contact occupancy in terms of hydrophobic or hydrophilic interactions are shown. During the projected 100 ns MD production run, nine residual contacts were established. Throughout the projected simulation, five molecular interactions remained until the end of the simulation—while the number of contacts increased, but diminished with time. The Tyr70, Asp72, Tyr121, Trp279, Asp285, Phe330, Phe331, and Phe334 in the peripheral active site gorge region interacted throughout the simulation. Despite this, Phe75, Gly80, Ser81, Trp84, Ser122, Ser286, Ile287, Gly335, and Ala336 displayed inconsistent molecular interactions—represented via the light orange color, meaning that a single contact was obtained during the simulation.

Furthermore, Asp72, Trp279, Asp285, and Tyr334 displayed a dark-orange band color representing high occupancy, with the maximum number of interactions throughout the simulation. It was further confirmed that approximately all probable positional geometries were achieved in these highly occupied H-bonds, as described in the aforementioned histogram plot. Despite this, at Asp72, Tyr121, Trp279, Phe331, and Phe334, a varied number of inconsistent contacts were exhibited throughout the simulated trajectory of each frame.

### 2.6. Molecular Investigation of Sarcorucinine-D’s Properties as an AChE Inhibitor

An in-depth study of Sarcorucinine-D’s properties was performed to obtain its geometrical and conformational differences. The Pre-MD run and post-MD output were used to correlate the properties of each atom of Sarcorucinine-D. Via detailed analysis of the noncompetitive AChE inhibitor Sarcorucinine-D by means of its deviation, fluctuation, and compactness via RMSD, RMSF, and Rg and surface area calculations in terms of its molecular surface, solvent-accessible surface, and polar surface areas, the MolSAs such as the SASA and PSA were computed. 

**Figure 6:**
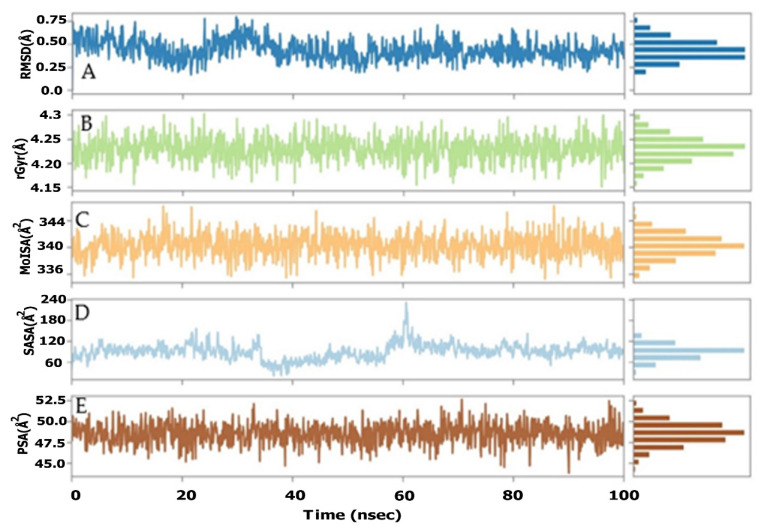
The ligand property analyses of the AChE–Sarcorucinine-D complex throughout the 100 ns simulation run trajectory. (**A**) Ligand RMSD; (**B**) Radius of Gyration; (**C**) Molecular Surface Area; (**D**) Solvent Accessible Surface Area; (**E**) Polar Surface Area.

The stability of the Sarcorucinine-D was further assessed by root mean square deviation (RMSD), which showed less fluctuation in the initial 10–35 ns of the MD runs. The Sarcorucinine-D RMSD showed a deviation of 0.4Å to 0.7Å, while the RMSD score was stable at 0.6Å over the projected simulation time, as determined in Figure 6A. The compactness of the Sarcorucinine-D, via the radius of gyration (rGyr), was further estimated. The rGyr of the Sarcorucinine-D showed consistency, with only minor fluctuation during the simulation run. The Sarcorucinine-D exhibited a rGyr value of between 4.2Å and 4.3Å (Figure 6B). Surface area calculations in terms of MolSA were also computed within a 1.4Å probe radius. The MolSA of the Sarcorucinine-D was found to be stable throughout the simulation, and fluctuated less at the 15 ns and 85 ns trajectory frames. The MolSA computed area was found to be in the range of 336Å^2^ to 344Å^2^, and the equilibrium achieved at around 342Å^2^ is visualized in Figure 6C. The accessible solvent surface area (SASA) was also estimated using a water (H_2_O) medium. Its value was also indicated by the sudden decrease near to 40 ns, with a SASA value of 50Å^2^; furthermore, it gradually rose to 60 ns with a deviation of 240Å^2^. After some time, the SASA stabilized and persisted at 120Å^2^. The SASA value ranged from 60 to 240Å, as illustrated in Figure 6D. The polar accessible solvent surface area, influenced by polar electronegative elements such as oxygen and nitrogen atoms, was also calculated for the Sarcorucinine-D molecule. The polar surface area lay in the range of 45Å^2^–53Å^2^ throughout the simulation. Visible fluctuations were observed at the 46 ns to 65 ns simulation period, while the equilibrium was attained at around 51Å^2^, as shown in Figure 6E. The aforementioned Sarcorucinine-D properties suggested that few fluctuations were spotted in the early or intermediate MD run of the 100 ns projected time. This distinctly indicates the stability of Sarcorucinine-D in the plausible binding of the AChE gorge cavity.

### 2.7. Pre- and Post-MD Results

The pre-MD and post-MD simulation results demonstrated that Sarcorucinine-D diffused in the PAS region of AChE. We compared molecular interactions in the static and flexible mode via molecular docking and molecular dynamics simulation approaches. The peripheral anionic site aromatic residues Tyr70, Trp279, and Tyr334 showed hydrophobic or pi–pi stacking contacts with Sarcorucinine-D in the pre-and post-MD binding mode analyses. Furthermore, the CAS residue Phe331 showed consistency and formed hydrophobic contacts in both the molecular docking and molecular dynamics simulations. Subsequently, Trp84, the PAS residue, and the CAS residue Asn85 showed hydrophobic contacts in the docking mode, and these interactions were not observed during the 100 ns simulation time. New molecular interactions with Asp285 evolved as a hydrogen bond interaction with Sarcorucinine-D during MD, but after some time, this hydrogen bond interaction converted into a hydrophobic interaction. The active site of the AChE enzyme was comprised of the CAS and PAS, which are the closest portion and the top position of the gorge region, respectively. Trp8 and Phe331 from the CAS and Tyr70, Trp279, and Tyr334 from the PAS were critical residues for interactions with target molecules. The most prominent consistency observed during MD was that of the aromatic ring of Phe331 towards the active site. As a result, the gorge’s bottleneck narrowed much more than it does in its natural state. The MD simulation study demonstrated that Sarcorucinine-D contacts with the target protein’s active site amino acid residues lasted for the majority of the simulation duration, forming a stable complex. 

### 2.8. Antispasmodic and Ca^2+^ Channel-Blocking (CCB) Properties

**Figure 7:**
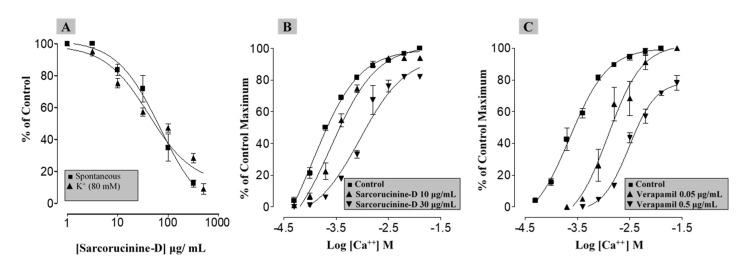
Graphs showing the gastrointestinal relaxant effect of Sarcorucinine-D in jejunal segments taken from a rabbit. (**A**) Curves represent the suppressant effect of the compound on spontaneous/natural contractions of jejunal tissues and on sustained contractility obtained using high K^+^ (80 mM; values shown are mean ± SEM, *n* = 3). Concentration–response curves exhibit the suppressant effect of increasing concentrations of (**B**) Sarcorucinine-D and (**C**) verapamil when tested against Ca^2+^ concentration–response curves obtained in a Ca^2+^-free medium in the jejunal tissues (values shown are mean ± SEM, n = 3).

Antispasmodic medications work by suppressing smooth muscle spasms in the gastrointestinal system. Anticholinergic characteristics are present in the most commonly used antispasmodics. To analyze its anticholinergic effects, Sarcorucinine-D was also studied for possible smooth muscle contractility-modulating effects in isolated rabbit jejunum. This preparation was selected because it can contract on its own by default, without using any agonist, which particularly helps in the pharmacological testing of new compounds with smooth muscle relaxant activity [26]. However, smooth muscle stimulants can also be tested, and the stimulant effects can be compared to spasmogenicity caused by standard spasmogenic agents. Sarcorucinine-D showed a concentration-dependent (3–300 µg/mL) suppression of jejunal contractility (EC_50_ = 0.07 µg/mL), clearly showing spasmolytic activity. Figure 7A graphically illustrates the spasmolytic effect of Sarcorucinine-D on spontaneous jejunal contractions.

The contractility in rabbit jejunum and other smooth muscle cells reflects the availability of cytoplasmic free Ca^2+^. This Ca^2+^ facilitates actin and myosin interactions, resulting in muscle contraction [27]. The earlier elevation of intracellular Ca^2+^ directly resulted from Ca^2+^ influx into a cell via the voltage-dependent Ca^2+^ channels (VDCs) or its release from the intracellular Ca^2+^ stores. These consistent cycles of depolarization and repolarization, with Ca^2+^ influx via VDCs, bring forth the typical spontaneous behavior of the intestine seen in our control experiments [28]. Sarcorucinine-D was able to suppress this contractile behavior of jejunal smooth muscles, possibly targeting Ca^2+^ release from intracellular Ca^2+^ stores or via inhibition of the entry of Ca^2+^ through the VDCs. To confirm this CCB activity, two different sets of experiments were performed. First, the effect of increasing concentrations of Sarcorucinine-D was tested on a high concentration of K^+^ (80 mM)-induced spasmogenicity. K^+^ (80 mM) contractions are a result of the influx of Ca^2+^ into the cells via VDCs; thus, suppression of K^+^ is likely via the inhibition of VDCs [29]. Sarcorucinine-D exhibited suppression (3–500 µg/mL) of this sustained contractility in the tissue (Figure 7A), with an EC_50_ value of 0.04 µg/mL. This showed that the compound acted via the blocking of VDCs, as is characteristic of a CCB, as substances that can inhibit K^+^-induced contractions are considered to be CCBs [24]. Following this preliminary finding of a CCB-like effect, this action was confirmed in a second set of experiments. The activity of Sarcorucinine-D was compared with that of a standard CCB, verapamil [30]. Both Sarcorucinine-D (10–30 µg/mL, Figure 7B) and verapamil (0.05–0.5 µg/mL, Figure 7C), in increasing concentrations, displaced the CaCl_2_ curves to the right. These curves were constructed in a Ca^2+^-free medium; this confirmed the CCB activity of sarcorucinine via the blocking of VDCs. 

ChE inhibitors elevate the concentration of acetylcholine at the cholinergic synapse, leading to muscle contraction. Apparently, this finding is contradictory to the spasmolytic effect manifested by Sarcorucinine-D in the rabbit jejunum. However, this action has been observed in many reported AChE inhibitors, which exhibit this behavior in vivo. Verapamil, which is a standard Ca^2+^ channel blocker, is one example.

Sarcorucinine-D was proposed to have both specific Ca^2+^ channel-blocking and ChE inhibitory activities. The Ca^2+^ channel blocking activity was manifested more prominently in the experimental system with isolated tissues. The diffusion of Sarcorucinine-D may be distorted by the presence of diffusion barriers, and its AChE inhibitory activity may also be affected by local changes in pH as the hydrolysis of acetylcholine proceeds [31]. Moreover, ChE inhibitors, βA aggregation blockers, and Ca^2+^ channel blockers are important AD drug candidates. Some calcium channel blockers, such as sabeluzole and nimodipine, are being tested in clinical trials as AD treatments [32,33].

### 2.9. Cytotoxicity Evaluation

Sarcorucinine-D was subjected to cytotoxicity evaluation using an MTT test. The compound was found to produce a toxic effect on MDBK (IC_50_ 2.24 μg/mL) when incubated for 72 h. No cytotoxic effect was observed for Sarcorucinine-D when incubated in a concentration range of 0.019–1.56 μg/mL for the same period (72 h).

This study’s results pinpoint Sarcorucinine-D as an exciting lead for anti-AD drug development, being a noncompetitive ChE inhibitor and Ca^2+^ channel blocker.

## 3. Material and Methods

### 3.1. Enzyme Inhibition Assays

AChE enzyme (*Torpedo californica*) and BChE enzyme (horse serum) and all the other chemicals were obtained from the Sigma-Aldrich Corporation (3050 Spruce Street, St. Louis, MO, USA). The enzyme activity was measured in vitro via a modified spectrophotometric method developed by Ellman et al. [34]. Acetylthiocholine iodide (ATCh) and butyrylthiocholine chloride (BTCh) were used as substrates for the AChE and BChE enzymes, respectively. Ellman reagent, i.e., 5,5-dithiobis (2-nitro) benzoic acid (DTNB), was used to produce the chromogenic indicator for the measurement of the ChE activity using a SpectraMax microplate spectrophotometer (Molecular Devices, CA, USA), as reported previously [18].

To test the selectivity of the compound, Sarcorucinine-D was also tested for the inhibition of several other enzymes such as phosphodiesterase, α-glucosidase, β-glucuronidase, and urease enzymes. The assays of these enzymes are reported elsewhere [35,36,37,38]. 

### 3.2. Estimation of Inhibition and Kinetic Parameters

The IC_50_ values (the test compound concentrations that inhibited substrate hydrolysis by 50%) were determined spectrophotometrically by measuring the effects of increasing concentrations of Sarcorucinine-D on the enzymes’ activity. The IC_50_ values were calculated using the EZ-Fit program (Perrella Scientific Inc., Amherst, MA, USA). Lineweaver–Burk plots [39], Dixon plots [40], and their secondary replots were used to observe the effects of the compound on the values of K_m_ and V_max_. Dixon plots, Lineweaver–Burk plots, and the secondary replots of Lineweaver–Burk plots were used to determine K_i_ values using initial velocities [39]. Initial velocities were attained over ATCh concentrations that ranged between 0.1 and 0.4 mM, and BTCh concentrations of 0.05 and 0.2 mM. The assay conditions were the same as the spectrophotometric assay procedure described earlier, except that fixed concentrations of the compound were used. The graphical analysis of the Dixon plots, Lineweaver–Burk plots, and their secondary plots was used to determine the inhibition type. 

### 3.3. Molecular Ligand–Protein Docking

The three-dimensional model of arcorucinine-D was constructed using the Dundee PRODRG2 Server [41]. This model was docked in the active-site gorge of *Torpedo californica* AChE (PDB ID; 7B2W) using Auto-Dock software [42]. The ligand was considered as a defined ligand-specific torsion tree with flexibility. The AChE structure was asserted as being rigid, and the grid was centered at the active-site gorges of the AChE, with dimensions of 70 Å × 70 Å × 76 Å and a grid spacing of 0.364 Å. An Auto-Dock algorithm with pre-calculated maps of each atom of the ligand and a pre-defined electrostatic potential was utilized. The auto-grid algorithm implemented in Auto-Dock predicted the binding energy, via its ligand conformation and contribution, of each atom of a specified element with the specified grid point in the vicinity of the receptor. Hydrogen atoms were added, and Gasteiger–Marsili charges were calculated using Auto-Dock Tools (https://ccsb.scripps.edu/mgltools, accessed on 8 May 2022). Docking results were analyzed with Biovia Discovery Studio visualizer 2021, WebLab ViewerPro v.4.0 [43], LIGPLOT v.4.5.3 [44], and LPC software [25].

### 3.4. Molecular Dynamic Simulation Set-Up

The analyzed docked complexes of the AChE–Sarcorucinine-D complex were subjected to molecular dynamics simulation. Desmond software was used to proceed with the 100 ns timescale of the production run. MD simulations of the sarcorucinine were carried out to explain the Sarcorucinine-D mode of inhibition in both ChE enzymes. The topologies of both ChEs receptors were generated, and an SPC model for solvation with a defined periodic boundary condition of 1.0 nm was applied to establish an aqueous medium. Additionally, both solvated ChE receptors were further subjected to the addition of Na^+^ or Cl^−^ ions for neutralization.

Furthermore, the pressure and temperature were retained at 1 atm and 300 K, as described in the Parrinello–Rahman algorithm and Nose–Hoover temperature coupling method. Moreover, the minimization and relaxation processes were executed by the NPT ensemble. The MD simulation of the 100 ns production run was attained with an interval period of 100 ps. Subsequently, the production run trajectories were evaluated by executing the simulation interaction diagram (SID) module implemented in the Desmond Schrödinger package. The following MD analytical parameters were computed, such as the protein deviation, fluctuation, compactness, hydrogen bonding, and their occupancies. Additionally, ligand properties during the run trajectories were also computed to record the alterations providing the optimum physiological conditions. 

### 3.5. Antispasmodic Activity in Isolated Rabbit Jejunum

Being an inhibitor of AChE, the effect of Sarcorucinine-D on muscular junctions was tested on isolated jejunal sections of the small intestines of rabbits, which contract naturally without the use of any pharmacological agent [45]. Local animals of around 1 Kg in weight were acquired, irrespective of gender. These were housed in the lab’s animal facility. All use of animals and their tissues complied with the rulings of the European Community guidelines, EEC Directive 86/609/EEC. The animals had access to water, as needed, while they underwent fasting for a day before the commencement of experimentation. Rabbits were sacrificed by cervical dislocation, and the jejunal section of the small intestine—around a couple of cm long—was obtained. These tissue segments were hung in an organ bath filled with a physiological salt solution, Tyrode’s, that was maintained at 37 °C and bubbled with carbogen gas. The muscular movements of the tissues were monitored and recorded with the help of transducers and student oscillographs from Harvard Apparatus, Holliston, MA. Isolated smooth muscles were left to stabilize in the above-mentioned setting for half an hour. The spontaneous contraction of rabbit jejunal tissues provides an essential method for screening putative gastrointestinal smooth muscle relaxant agents. Later, standard agonists and the test compound were introduced, once the tissues had stabilized. 

### 3.6. Determination of Calcium Antagonist Activity

Once a test substance exhibited smooth muscle relaxant activity, it was a candidate for testing for potential CCB action. For this determination, a high dose (80 mM) of K^+^ was utilized to induce sustained contractility in jejunal tissues [46]. When the contractility was sustained and continuous, Sarcorucinine-D was added to this to see if the compound could exhibit some sort of suppression or relaxant effect [47]. This potential relaxant effect was quantified as a percent of the standard maximal contraction produced by K^+^. 

Another set of confirmatory experiments was also performed to be sure of any potential CCB activity. For this, the regular Tyrode’s physiological salt solution in which the tissues were originally immersed was removed. The baths were filled with a Ca^2+^–free Tyrode’s solution with EDTA (0.1 mM). The tissues were left in this solution for half an hour. Later, another replacement was carried out using K^+^-rich and Ca^2+^-free Tyrode’s solution. The tissues were again left to stabilize for half an hour, and then, the control concentration–response curves of Ca^2+^ were constructed and recorded. These control Ca^2+^ concentration–response curves were duplicated after pretreatment of the tissues for one hour with different concentrations of Sarcorucinine-D. Any possible CCB effect of Sarcorucinine-D would tend to displace the Ca^2+^ concentration–response curves to the right-hand side. The effects of Sarcorucinine-D were compared to those of a standard CCB, verapamil.

### 3.7. Cytotoxicity Evaluation

The MTT (3-[4,5-dimethylthazol-2-yl]-2,5-diphenyltetrazolium bromide) test was performed using MDBK adherent cells. Cells (1 × 10^4^) were incubated with serial dilutions of Sarcorucinine-D (0.195 to 50 µg/mL) for three days. Cell viability and IC_50_ (concentration of compound resulting in 50% inhibition of cell growth or reducing cell survival by 50%) were measured after 72 h, as described previously [48].

### 3.8. Statistical Analysis

All assays conducted in this study were performed in triplicate. The graphs of enzyme inhibition were plotted with the GraFit program [49]. Values for the correlation coefficient, slope, intercept, and their standard errors were obtained by linear regression analysis using the same software. The correlation coefficient for all the lines of the graphs (Figure 1) was >0.99. 

The antispasmodic data were analyzed and plotted using GraphPad Prism software [50]. The results were expressed as mean ± standard mean error (SEM). 

## 4. Conclusions

In conclusion, in vitro enzyme inhibition kinetics demonstrated that Sarcorucinine-D inhibits the two cholinesterases (AChE and BChE) noncompetitively in the micromolar range. The enzymes’ activities were investigated experimentally and the molecular bases of interactions between the inhibitor and the enzymes were explored experimentally (inhibition kinetics) and theoretically (MD simulation and docking). In silico docking and MD simulation studies predicted interesting hydrophobic interactions and hydrogen bonds with the residues of the active-site gorge of AChE. The effect of Sarcorucinine-D on muscular junctions revealed that the compound induced gastrointestinal relaxant action when tested in vitro on smooth muscle preparations. Upon testing, it was found that this relaxant effect was via the blocking of VDCs. The safety of the compounds was confirmed by an in vitro cytotoxicity assay against MDBK cells. 

Therefore, Sarcorucinine-D could be considered a potential lead molecule for Alzheimer’s disease based on its properties as a noncompetitive AChE inhibitor and a Ca^2+^ channel blocker. However, further in vivo and preclinical studies would help provide further insights into this lead compound’s pharmacological properties.

## Figures and Tables

**Table 1:** Experimental kinetic parameters of in vitro inhibition of *Tc*AChE and BChE by sarcorucinine-D.

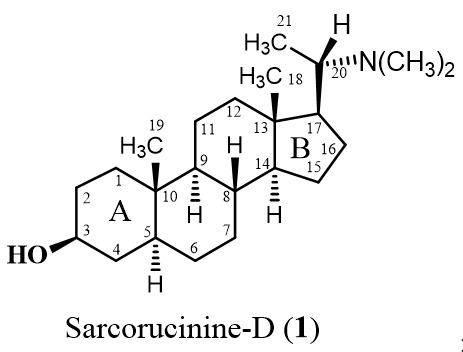						
**Compound**	**Acetylcholinesterase**			**Butyrylcholinesterase**		
	**IC_50_ (µM)** **^a^**	**K_i_^b^ (µM)** **^a^**	**Inhibition**	**IC_50_ (µM)** **^a^**	**K_i_ (µM)** **^a^**	**Inhibition ^c^**
Sarcorucinine-D	42.9 ± 3.13	103.3 ± 6.67	NC	8.87 ± 0.56	4.66 ± 0.3	NC
Tacrine	0.021 ± 0.002	0.23 ± 0.02	MT	0.051 ± 0.005	0.025 ± 0.003	MT
Galanthamine	0.45 ± 0.02	0.19 ± 0.01	MT	39.1 ± 0.032	32.0 ± 0.33	NC

^a^ Experimental IC_50_ and K_i_ values (mean ± SEM of three experiments). ^b^ K_i_ was calculated from Dixon plot, Lineweaver–Burk plot, and its secondary replots. ^c^ NC = non-competitive inhibition MT = mixed-type inhibition.

**Table 2:** Structure and interactions of Sarcorucinine-D with *Tc*AChE predicted by LPC and LIGPLOT.

Binding energy (kcal/mol) ^a^		−12.73
Estimated K_i_^b^		4.66 × 10^−10^
Compl. values ^c^		0.53
Solv. accessible surface ^d^		45.1/564.4
Residues involved in specific contacts with Sarcorucinine-D ^e^	polar contacts (Enz-Inh.)	Glu199 (O…O-H) Inh. (2.7Å) Tyr130 (O-H…O) Inh. (2.8Å)
	non-polar contacts	Tyr70; Asp72; Trp84; Ser122; Leu127; Trp279; Phe290; Phe330; Phe331; Tyr334; His440; Ile444
	destabilizing contacts	Asp72; Trp84; Gly117; Gly118; Ser122; Gly123; Tyr130; Glu199; Ser200; Phe330; Tyr334; His440; Ile444

^a^ Binding energy was calculated by by Auto-Dock ^b^ Inhibition Constant (Ki) was estimated by Auto-Dock (Temp. = 298.15 K) ^c^ The normalized complementarity function is calculated by LPC as CF = S_l_-S_i_-E; where S_l_ is the sum of all surface areas of legitimate atomic contacts between ligand and receptor, S_i_ is the sum of all surface areas of illegitimate atomic contacts, and E is a repulsion term (see Ref. [24]). ^d^ Solvent accessible surface is shown for complexed/uncomplexed inhibitor. ^e^ Atoms of all amino acids can form more than one contact (either stabilizing or destabilizing) with atoms of the ligand. Polar contacts are hydrogen bonds; non-polar contacts refer to hydrophobic–hydrophobic contacts, while destabilizing contacts are mainly hydrophobic–hydrophilic in nature.

## Sample Availability

Samples of the compound are not available from the authors.
